# Comparison of hook plate versus T-plate in the treatment of Neer type II distal clavicle fractures: a prospective matched comparative cohort study

**DOI:** 10.1186/s13018-022-03261-8

**Published:** 2022-07-30

**Authors:** Mehdi Teimouri, Hadi Ravanbod, Amirhosein Farrokhzad, Jamal Sabaghi, Seyed Peyman Mirghaderi

**Affiliations:** 1 Department of Orthopedic Surgery, Ayatollah Kashani Hospital, Isfahan University of Medical Sciences, Isfahan, Iran; 2 Department of Orthopedic Surgery, Al-Zahra Hospital, Isfahan University of Medical Sciences, Isfahan, Iran; 3 Joint Reconstruction Research Center, Imam Khomeini Hospital Complex, Tehran University of Medical Sciences, Tehran, Iran; 4 Students’ Scientific Research Center (SSRC), Tehran University of Medical Sciences, Tehran, Iran

**Keywords:** Bone plates, Clavicle, Distal clavicle fracture, Hook plate, Internal fixators, T-plate

## Abstract

**Background:**

This study aimed to compare the clinical and radiological outcomes of distal clavicle fracture fixation with a hook plate versus the standard non-locking T-plate for unstable Neer type II fractures.

**Methods:**

A prospective matched cohort study including two groups of hook plates and T-plates fixation was conducted in our two tertiary trauma centers. Patients with distal clavicle fractures Neer type II were assessed for union and the Constant-Murley score (CMS) at 1-, 3-, and 6-month follow-ups. Inadequate radiographic consolidation > 6 months after surgery was defined as non-union. Subscales of CMS_pain_, CMS_activities of daily living_, CMS_range of motion (ROM)_, and CMS_strength_ were also compared between groups. According to recommendations, the implant was removed after union confirmation in the hook plate at a planned second surgery.

**Results:**

Sixty consecutive patients were enrolled: 30 in the T-plate group and 30 in the hook plate group. CMS showed similar functional outcomes for T-plates and hook plates at all follow-ups (Month 6: 92.0 vs. 91.7, *P* = 0.45). However, on the month 1 follow-up, the T-plate group scored higher than the hook plate group for ROM and pain (CMS_pain_ = 13.0 vs. 12.3, *P* = 0.03; CMS_ROM_ = 35.2 vs. 33.2, *P* = 0.002). Despite this, Pain, ROM, and other CMS domains were comparable between groups (*P* > 0.05). The mean time to union was 2.5 + 1.4 months for the T-plate group and 2.3 + 1.6 months for the hook plate group (*P* = 0.44). There was one fixation failure in each group and one periprosthetic fracture in the hook plate group (two revisions for the hook plates and one for T-plates, *P* = 1.00). Non-union and other complications were not observed.

**Conclusion:**

Both surgical approaches resulted in full recovery and good function. However, in the hook plate group ROM and pain scores were lower at 1 month. Standard non-locking T-plates are a viable alternative to hook plates with low cost and promising outcomes for treating displaced distal clavicle fractures.

## Introduction

Distal clavicle fractures are among the most common injuries in adults, accounting for 2.6–5% of all fractures and 21% of clavicle fractures [1–5]. It constitutes up to 45% of clavicle non-unions and can result in severe disability if not treated adequately; therefore, unstable distal clavicle fractures should receive appropriate surgical treatment [2, 6, 7]. Distal clavicle is anatomically made of metaphyseal bone with a small distal fragment. This makes it difficult to achieve stable fixation and early motion [8]. Several surgical devices have been used to fix this fracture in recent years, including locking anatomical plates, hook plates, T-shape plates, trans-acromial pinning, double plates, and tension band wiring. However, there is no gold standard yet [2, 9–13].

Hook plates are widely used and help create a stable lever that elevates the acromion and pushes the clavicle downward to hold it firmly in place, negating movement of the broken part while not interfering with clavicle rotation [12, 13]. Hook plate fixation is a suitable method with an acceptable union rate when the remaining distal clavicle fragment is small and cannot be adequately fixed [11]. This method has disadvantages, including the need to remove the implant to achieve full ROM and eliminate the unpleasant feeling of having an external device attached [14]. Also, using the subacromial space for clavicular hook plating may cause adverse effects, such as rotator cuff tear and subacromial impingement, causing pain and stiffness of the shoulder that takes time to subside [12, 14–18].

Another potential method is fixing with T-plates, a 3.5-mm low-profile titanium plate, introduced by Kalamaras et al. in 2008 [8, 19]. T-plate fixation has demonstrated high union rates, good function, and low complication rates [19–22]. The plate design enables the insertion of three screws into the small distal fragment, providing stability [22]. A lack of evidence exists for a direct comparison between T-plate and Hook fixation of a distal clavicle fracture [11].

Controversy still exists regarding the effectiveness and complications of surgical treatment options for unstable distal clavicle fractures. This study aimed to compare the clinical and radiological outcomes of unstable Neer type II distal clavicle fracture fixation with a non-locking hook plate versus a standard non-locking T-plate. We hypothesized that the T-plate fixation has comparable union rates and functional outcomes at a six-month follow-up.

## Methods

### Study design and setting

**Fig. 1:**
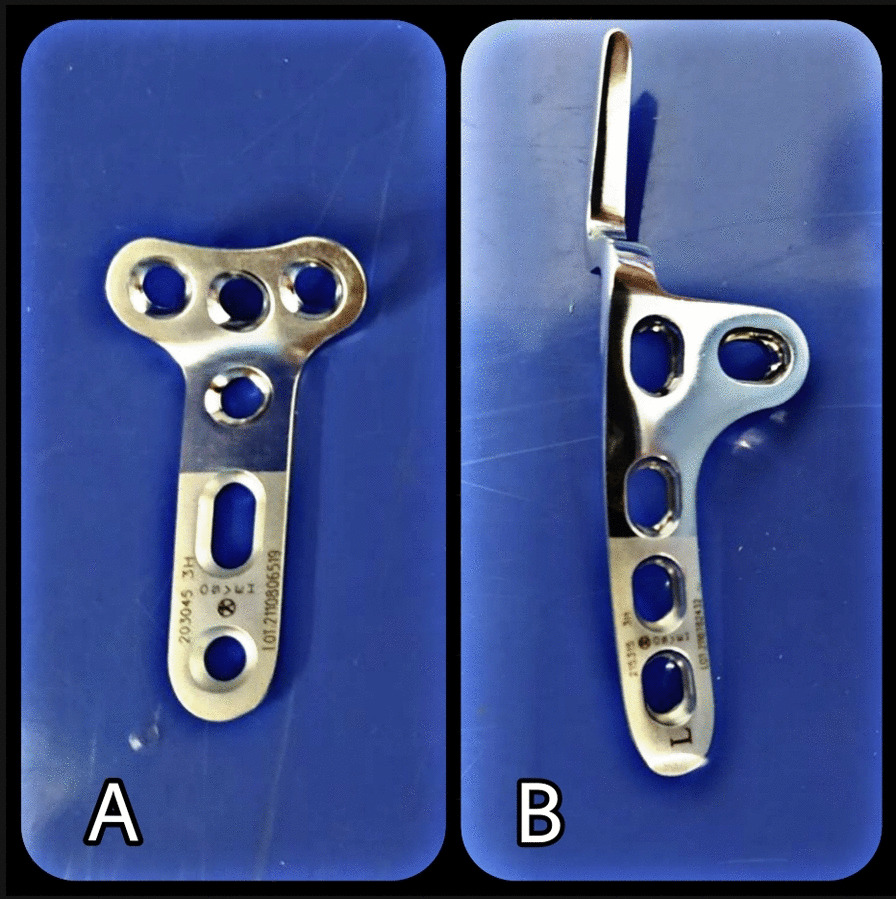
Two fixation devices investigated in the study, **A** T-plate, **B** hook plate

Between March 2019 and January 2021, a prospective matched cohort study of patients with distal clavicle fractures Neer type II was conducted in two tertiary trauma centers (Ayatollah Kashani hospital and Al-Zahra, Isfahan, Iran). In the included patients, surgical fixation was needed, and they were fixed with two different instruments: non-locking hook plates and standard non-locking T-plates (Fig. 1). The institutional review board (IRB) of Isfahan university of medical sciences approved the study’s protocol and declared there is no ethical concern (Approval ID: IR.MUI.MED.REC.1399.705). All patients signed a written informed consent statement and voluntarily participated in the study.

### Surgical technique and postoperative rehabilitation

Patients were operated on by two skilled orthopedic surgeons (M.T and H.R) using either a hook plate or a standard T-plate. Both were titanium 3- or 4-hole 3.5-mm nonlocking plates (Fig. 1). All the surgeries were performed under general anesthesia and in a beach chair position. Through an anterosuperior approach and a ~ 6–8 cm incision, the distal fragment was exposed and reduced under visualization, while the acromioclavicular (AC) joint was preserved. For the hook plate, the hook was inserted under the acromion process. Whenever necessary, the clavicle part was contoured to fit the clavicle to ensure proper fixation. The fixation was completed with 3 screws at both the distal and proximal parts of the plate. For the T-plate, the T-shaped part and shaft plate were slightly flattened to conform to the distal and proximal fragments. Three screws were placed in the T-plate's distal and proximal parts for the final fixation. Fluoroscopy ensured that the screws did not penetrate the acromioclavicular joint. There was no repair of the coracoclavicular ligaments in either group.

Postoperative rehabilitation and management were same for the two groups. Immediately after surgery, patients should wear a sling arm for 2 weeks to protect their shoulder. Ideally, patients should mobilize their shoulder joint as soon as possible, especially if pain free. After two weeks, patients were encouraged to discontinue the sling and increase pendulum exercise up to 90° until 6 weeks. Active shoulder ROM began after 6 weeks post-operation. For hook plate patients, an abduction limit of 90° was prescribed until radiographic proof of healing to prevent implant-related adverse effects. It was not permitted to use the arm vigorously until six months following surgery. Active ROM exercises were begun for the elbow, wrist, and hand after recovering from anesthesia. At our center, supervised physiotherapy was not routine and no patients were sent to physical therapist. For pain management, only Acetaminophen 500 mg PRN was prescribed.

According to recommendations, the implant was removed after union confirmation in the Hook plate at a planned second surgery [12, 23, 24]. The plate removal is the standard procedure in our center. If the patient was satisfied with the hook plate and declined the second surgery, the hook plate remained in place. T-plates were not removed until they became symptomatic.

### Outcome measures and data collection

Patients were assessed at 1, 3, and 6 months after discharge. At this time, patients are evaluated by X-ray radiography and physical examination. Standard AP radiography is analyzed for union, and the Constant-Murley score (CMS) is used to assess shoulder function and pain. The definition of the bone union of fractures is the obliteration of the fracture line and bridge of the bony callus [25]. Inadequate radiographic consolidation > 6 months after surgery was defined as non-union. The CMS after six months was the primary outcome measure used to compare the two groups. This measurement includes objective and subjective elements and is scored on a 100-point scale. The scale consists of four domains: pain (15 points), activities of daily living (20 points), range of motion (40 points), and strength (25 points). CMS's minimal clinically important difference (MCID) was determined to be 10.4 [26–28]. All the complications assessed and documented during the study follow-up include non-union, fixation loosening, failure, infection, neurovascular damage, periprosthetic fracture, etc.

The physical examinations and functional assessments were conducted by an expert orthopedic resident (J.S, PGY-3) blinded to the study groups. A blind examination of the radiography couldn’t be conducted because of apparent differences in plate shapes between groups in the radiograph. Demographic information and comorbidities of the patient were retrieved.

### Participants, sample size, and inclusion criteria

The sample size was estimated using the two-mean comparison formula and based on the data related to the CMS: We considered 0.05 and 0.10 as the type I (α) and II (β) errors, respectively, while σ is the standard deviation of the variable in the population. In addition, µ1 and µ2 are the means of the investigated variables in the two groups. Using the CMS of Erdle et al. study [29] and considering *µ*_1_ = 92.2 and *µ*_2_ = 88.7 for the two groups and *σ* = 4.2, the minimum sample size was calculated to be 30 in each group (total sample size of 60 patients).

The inclusion criteria are 1. acute, unstable, isolated unilateral Neer’s type II distal clavicle fracture based on Craig's modification of Neer's classification [30], 2. age between 20 and 50, 3. ASA class I or II, and 4. the size of the clavicle's distal fragment allows the insertion of 3 screws in the distal part of the plate, to allow surgeons to choose either device. The exclusion criteria are 1. undisplaced fracture (Neer’s type I), 2. non-traumatic fractures, 3. concomitant fractures include coracoid, glenoid, acromion, scapula, and proximal humerus or any concomitant shoulder girdle injury, 4. an injury that induced neuropathy and neural complications (hindered clinical examination), 5. prior shoulder pathology and disability, 6. small distal fragment sizes that would limit insertion of three screws, and 7. not intended to participate or be illiterate to complete the follow-up and fill the forms. Patients were enrolled until the sample size was achieved and matched by sex and age (± 5 years).

### Statistical analysis

SPSS v.23.0 software (IBM SPSS Inc., USA) was used to analyze the data. Normality was assessed using the Shapiro–Wilk test. Student's t-test, Mann–Whitney test, and Analysis of Variance (ANOVA) were employed to compare continuous variables based on their normality. In addition, the chi-square and Fisher exact tests were used to compare the nominal variables. To compare the group scores at different times, the repeated measures ANOVA test was used. *P*-value < 0.05 (two-sided) was considered significant.

## Results

**Fig. 2:**
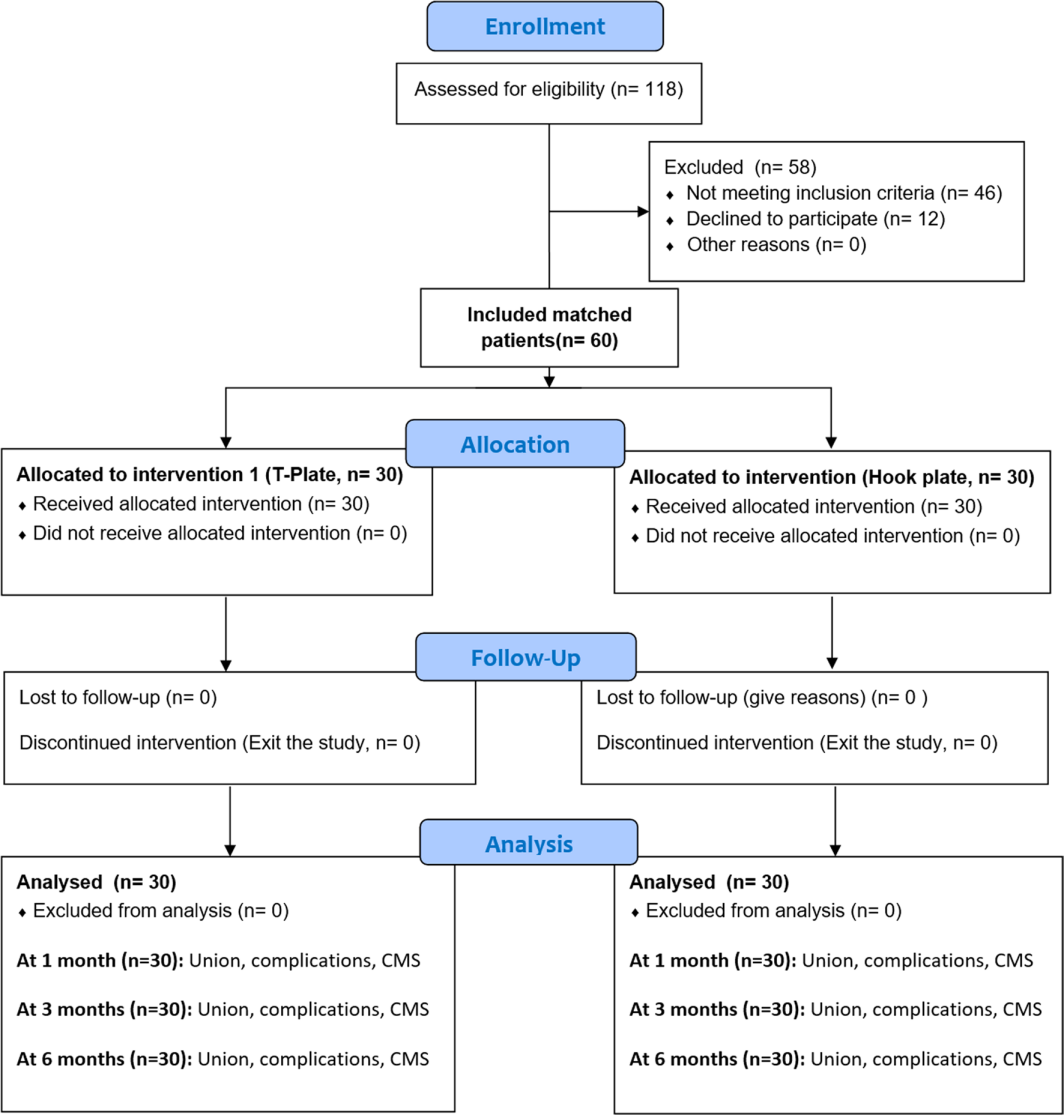
Flow diagram of patients’ enrollment and evaluations

**Fig. 3:**
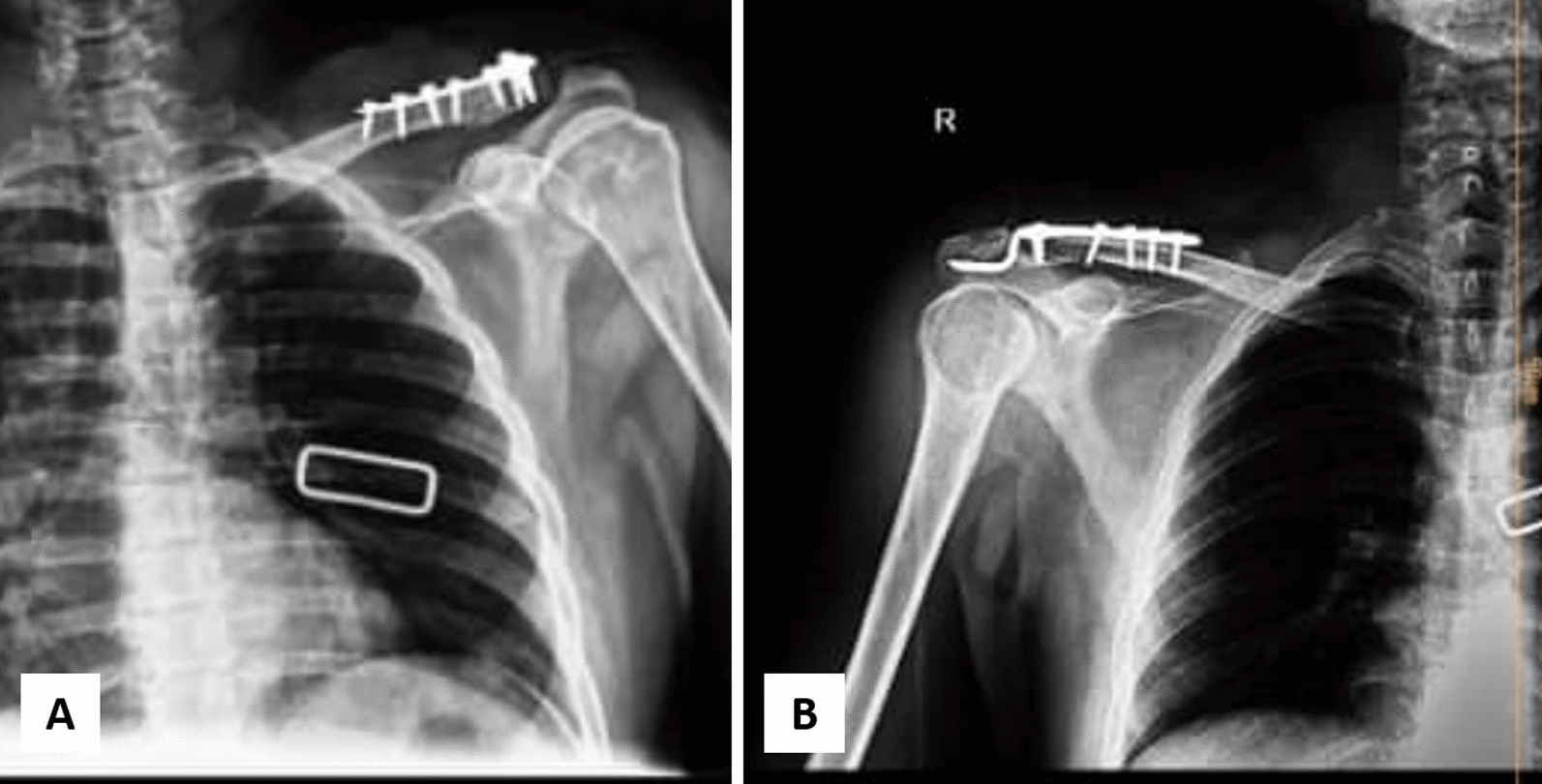
Distal clavicle fracture fixation using **A** T-plate and **B** hook plate

Sixty consecutive patients with Neer II (A + B) distal clavicle fractures were enrolled in the study, 30 in each of two groups that were matched for age and sex (Fig. 2): 30 in the Hook plate group and 30 in the T-plate group (Fig. 3). Both patient groups were similar in terms of demographics, comorbidities, side of injury, injury to the dominant arm, and smoking status (*P* > 0.05) (Table 1).

**Table 1:** Demographic characteristics (mean ± SD or *n*, %)

Demographics	T-plate	Hook plate	*P*-value
Number	30	30	
Sex (male: female)	23 (76.7%):7 (23.3%)	23 (76.7%):7 (23.3%)	1.0
Age, years	40.5 ± 12.0	41.0 ± 10.5	0.86
BMI	26.1 ± 4.7	25.5 ± 4.9	0.63
Side (right:left)	19 (63.3%):11 (36.7%)	21 (70.0%):9 (30.0%)	0.58
Dominant upper limb injury	20 (66.7%)	21 (70.0%)	0.78
Mechanism of injury			
Falling/sport	21 (70%)	23 (76.7%)	0.56
Traffic accident	9 (30%)	7 (23.3%)	
Days from injury to surgery (range)	1.1 ± 0.8 (0–3)	1.0 ± 0.7 (0–2)	0.81
Diabetes mellitus	2 (6.7%)	1 (3.3%)	1.0
Smoking	10 (33.3%)	8 (26.7%)	0.57
ASA class (I:II)	15 (50.0%):15 (50%)	18 (60%):12 (40%)	0.43

*BMI* body mass index, *ASA* American Society of Anesthesiologists Classification

**Fig. 4:**
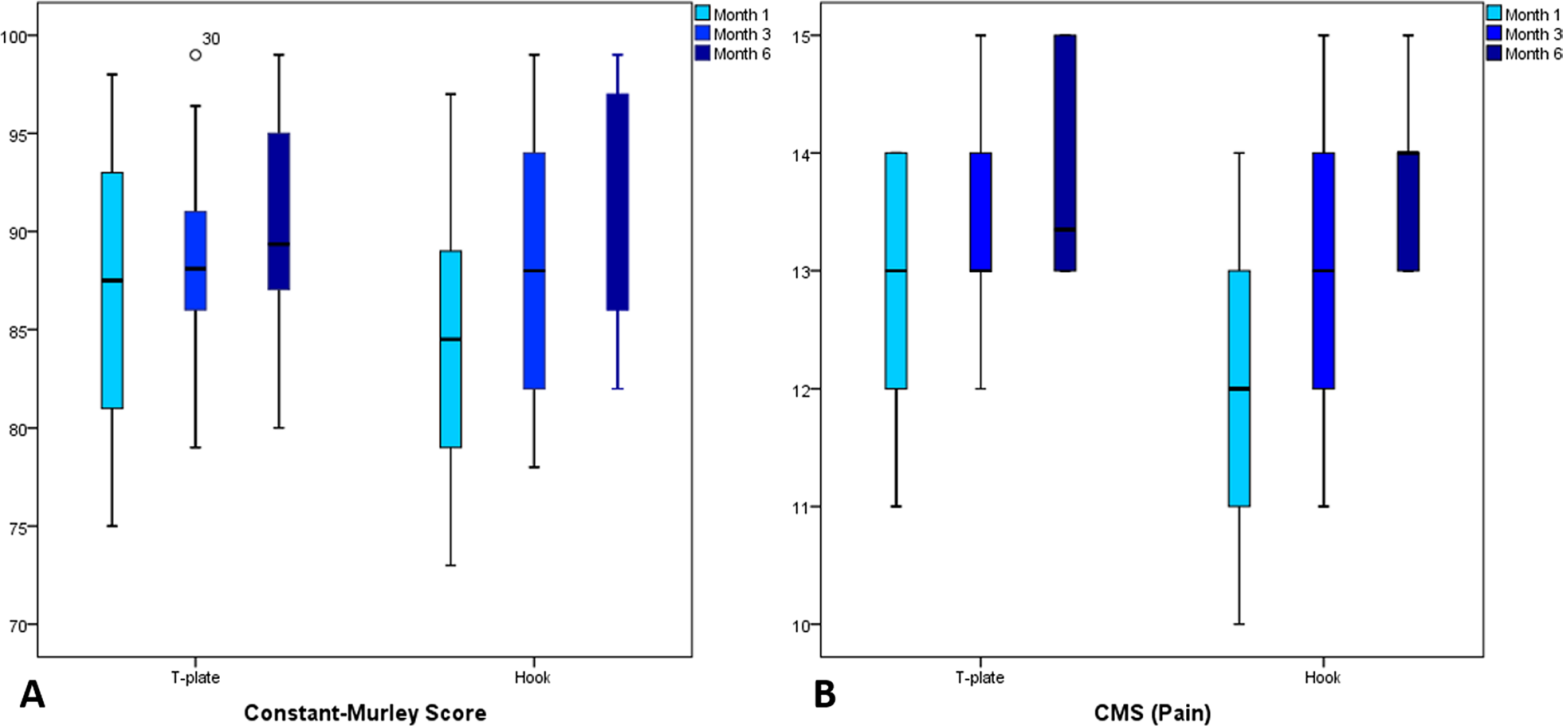
The comparison of **A** Constant-Murley scores (CMS) and **B** CMS_pain_ at different follow-up visits after surgery

**Fig. 5:**
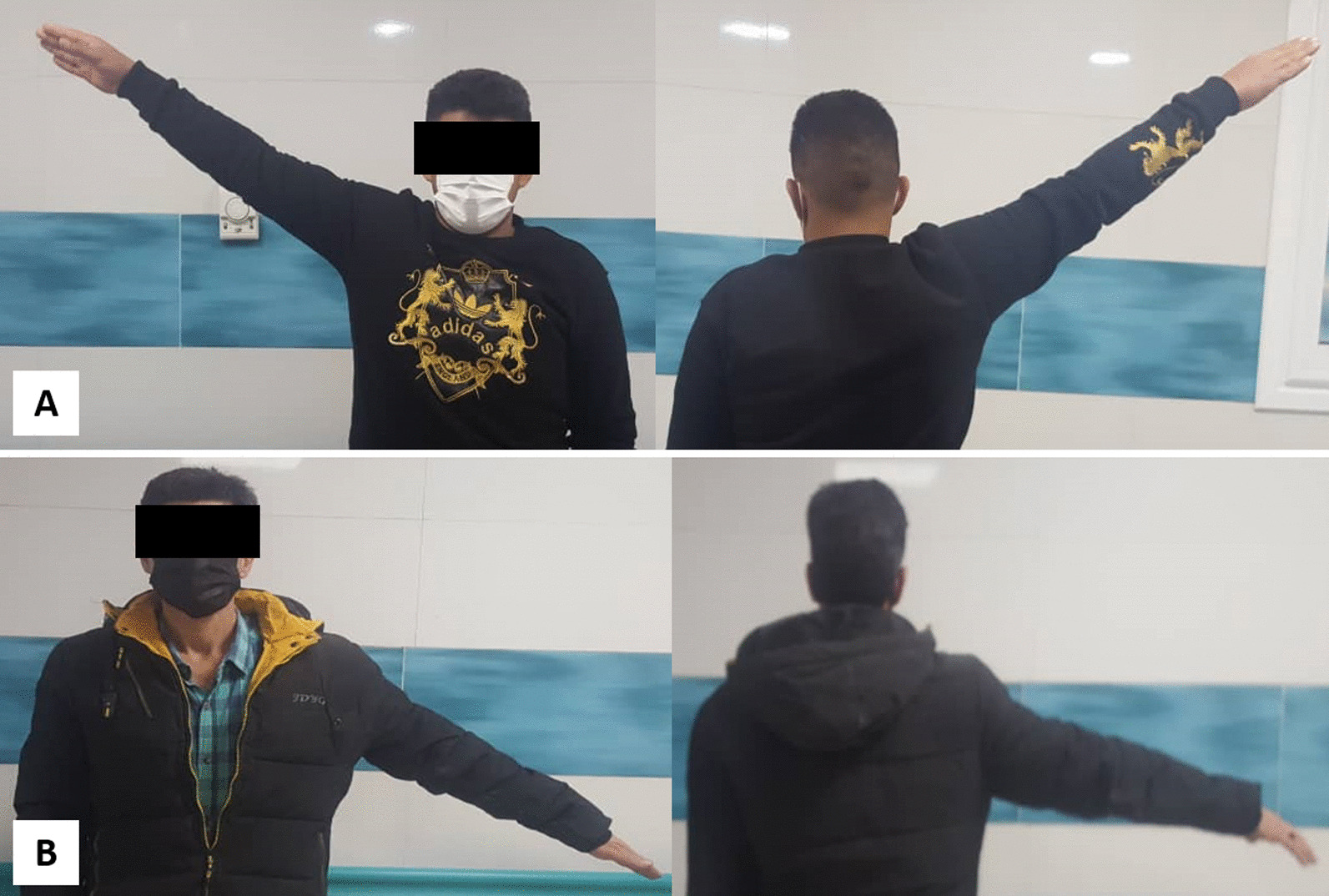
Comparison of abduction range of motion in the **A** T-plate, **B** and hook plate

Table 2 summarizes the study's outcomes. CMS showed similar functional outcomes between the two groups in the three follow-ups (*P* > 0.05) (Fig. 4a). However, the T-plate group has superior ROM and pain scores at the earliest follow-up than the Hook plate group (CMS_pain_ = 13.0 vs. 12.3, *P* = 0.03; CMS_ROM_ = 35.2 vs. 33.2, *P* = 0.002) (Fig. 4b) (Fig. 5). Pain and ROM scores were higher but not significantly in the T-plate following future follow-ups (*P* > 0.05). Both groups showed comparable results in other CMS domains, including activities of daily living and strength (*P* > 0.05). The union was assessed at 1, 3, and 6 months post-operation, and the mean time to union was 2.5 + 1.4 months for the T-plate group and 2.3 + 1.6 months for the Hook plate group (*P* = 0.44).

**Table 2:** Functional scores Constant-Murley score (mean ± SD or *n*, %)

Follow-up	Month 1			Month 3			Month 6		
Groups	T**-**plate	Hook plate	*P*-value	T**-**plate	Hook plate	*P*-value	T**-**plate	Hook plate	*P*-value
Constant-Murley score (CMS)	86.7 ± 6.0	84.1 ± 6.1	0.10	88.6 ± 4.7	88.7 ± 6.6	0.81	91.7 ± 5.4	92.0 ± 5.3	0.45
CMS_Pain_ (max. 15)	13.0 ± 0.9	12.3 ± 1.3	0.03*	13.3 ± 0.8	13.0 ± 1.1	0.27	13.8 ± 0.8	13.7 ± 0.7	0.78
CMS_Activities of daily living_ (max. 20)	17.1 ± 1.3	17.1 ± 1.3	0.98	17.5 ± 1.0	17.8 ± 1.5	0.27	18.2 ± 1.2	18.5 ± 1.2	0.41
CMS_Strength_ (max. 25)	21.4 ± 1.5	21.5 ± 1.5	0.73	22.2 ± 1.2	22.4 ± 1.6	0.49	23.0 ± 1.4	23.5 ± 1.4	0.21
CMS_Range of motion_ (max. 40)	35.2 ± 2.4	33.2 ± 2.4	0.002*	35.6 ± 1.9	35.5 ± 2.7	0.72	36.6 ± 2.2	36.2 ± 2.3	0.37
Union	11 (36.7%)	15 (50%)	0.29	28 (93.3%)	27 (90%)	1.0	30 (100%)	30 (100%)	1.0

*A significant difference between groups

**Fig. 6:**
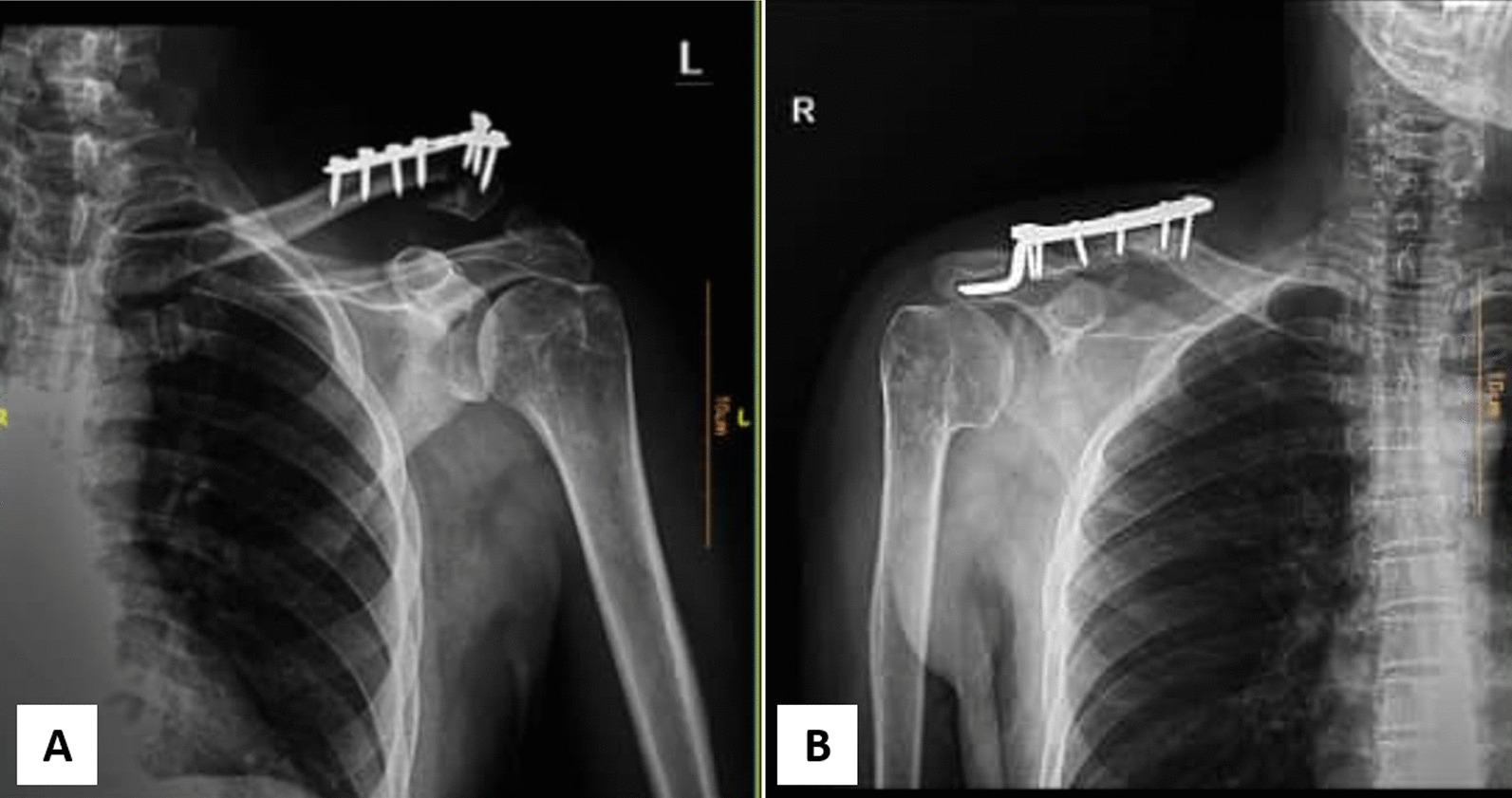
Fixation failure needs revision surgery in the **A** T-plate and **B** Hook plate groups

**Fig. 7:**
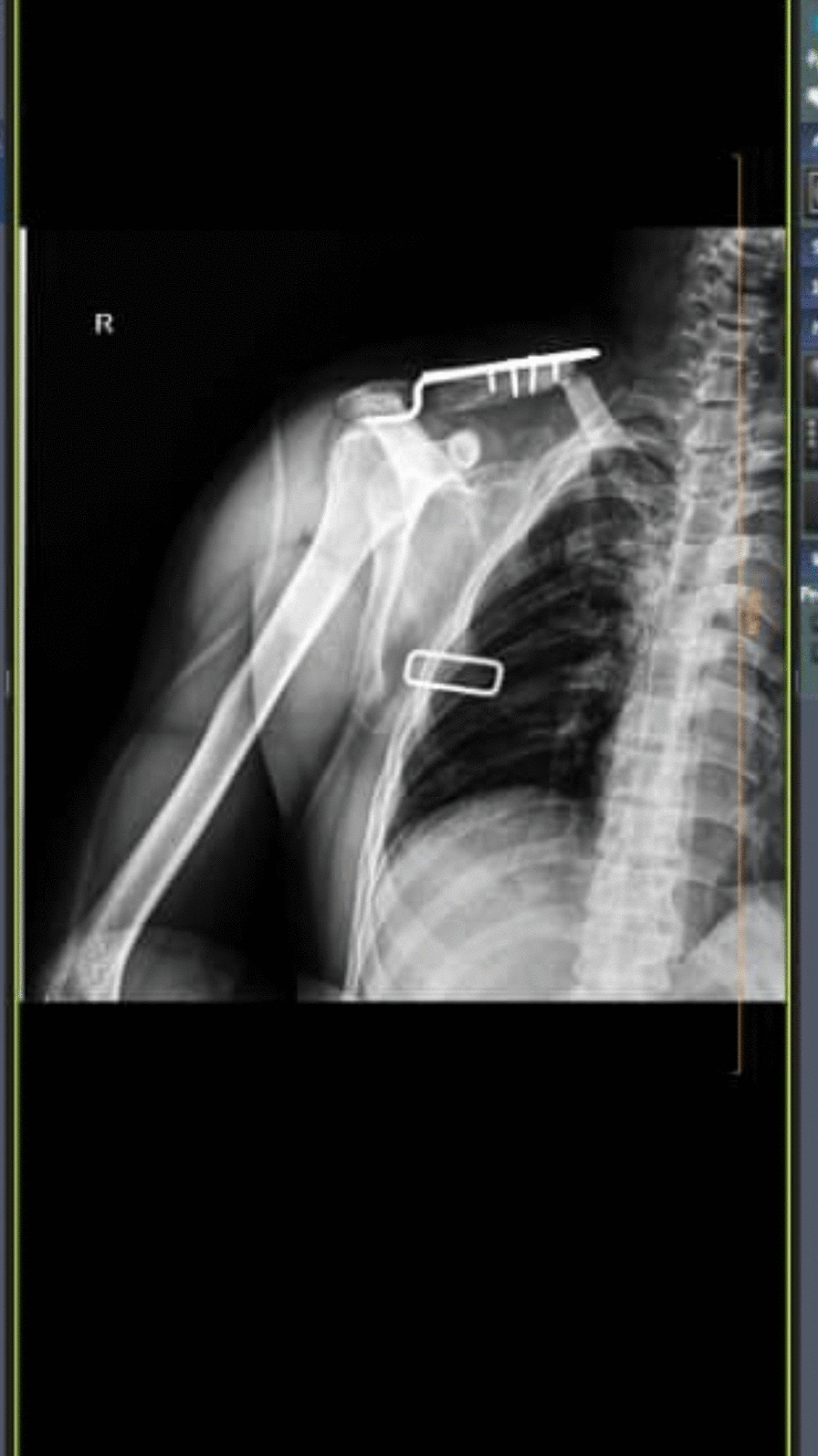
Periprosthetic fracture in hook plate fixation

Table 3 shows complication rates and reoperation rates across both groups. No intraoperative complications were observed. Regarding postoperative complications, both groups had one fixation failure (Fig. 6). A periprosthetic fracture occurred in one of the Hook Plate patients, who required reoperation (Fig. 7). Thus, 2 revision surgery performed in the hook plate group, while one was performed in the T-plate, and all proceeded with acceptable healing. There were no cases of non-union, surgical site infection, osteolysis, stiffness, or neurovascular damage.

**Table 3:** Surgical complication and reoperation in T plate and Hook plate

Complication and reoperation	T plate	Hook plate
Fixation failure (*P* = 1.0)	1 (3.3%)	1 (3.3%)
Surgical site infection	0	0
Non-union	0	0
Neurovascular damages	0	0
Peri-device fracture	0	1 (3.3%)
Acromial osteolysis	0	0
Adhesive capsulitis/stiffness	0	0
Need for revision (*P* = 1.0)	1 (3.3%)	2 (6.7%)

**Fig. 8:**
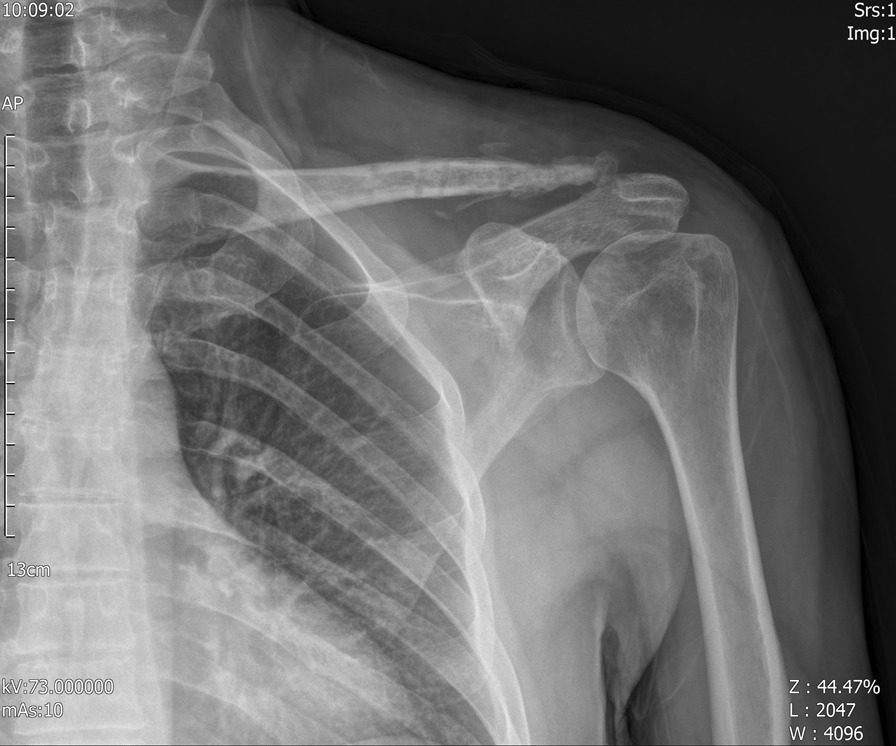
X-ray radiograph after plate removal in the Hook plate group

As a standardized procedure, hook plate removal was performed in all patients (Fig. 8), but in five who declined removal (83.33%). On average, hook plates were removed at 4.5 months (range: 3–6 months) after surgery. In the T-plate group, surgery for hardware removal was advised only in symptomatic patients, and thus no plates were removed by the 6-month follow-up.

## Discussion

The main findings of this study were that using T-plate fixation for the unstable Neer type II distal fracture has comparable good union and functional results compared to hook plating. Although both methods exhibited a low complication rate, using a T-plate is associated with better ROM and pain scores at 1 month. We believe that utilizing a standard non-locking 3.5-mm T-plate device for unstable and displaced distal clavicles is an affordable, novel, and effective method.

A recent meta-analysis of 1261 patients by Malik et al. comparing hook plate and superior plate for treatment of displaced distal clavicle fractures found that the hook plate had a significantly higher overall complication rate (32.7%) than the superior plate (12.7%, OR = 6.3). Hook plate revealed 11.3% acromial osteolysis, 2.8% peri-prosthetic fracture, 1.6% shoulder stiffness, 1.6% impingement, 1.2% infections, and 0.35% rotator cuff tear [31]. Using a superior plate alone caused 11.7% of implants to be removed due to pain or cosmesis and 0.45% infection. However, they showed a similar union rate (~ 97%) and CMS score [31]. Another recent meta-analysis comparing different internal fixation techniques has demonstrated that locking compression plates + coracoclavicular fixation is the most effective and has the least number of complications [4]. Meanwhile, hook plate and other techniques tended to result in lower functional scores and higher complications. Thus, although hook plates provided rigid fixation and high union rates, there are concerns about their complications and lower functional scores [32]. An additional meta-analysis revealed that CMS at 3 and 6 months for the distal clavicle locking plate is superior to clavicle hooks [32]. In agreement with them, we found that all participants had fully healed, but participants who had hook plates experienced more pain and limited ROM of the joint in the first month of follow-up. In the subsequent follow-ups, the two groups were comparable. Complications did not differ between the two groups.

The hook plate is designed to fit the clavicle and acromion anatomically, with the lateral hook nestled beneath the acromion posterior to the AC joint and the body attaching to the superior surface of the clavicle. With the help of the subacromial hook, the body of the plate acts as a depressor, gently pressing the medial fracture fragment down, facilitating bone union and early postoperative mobilization [23, 33, 34]. On the other hand, surgical complications associated with the hook plate were found to be more than plate and screw fixation [2, 4, 31]. Inflammation of the subacromial space, impingement of the rotator cuff, osteolysis of the acromion (27%), and periprosthetic fractures (22%) are all potential consequences of hook placement [1, 16]. The presence of all complications was reported to be even as high as 63% in the previous studies, and most of them were specific to the implant, such as AC joint arthrosis [29]. In this regard, Erdle et al. reported an inferior AC joint-specific score (Taft score) for the hook plate fixation rather than the plate, which could be caused by hook micromotions around the AC joint [29]. The hook plating did not result in osteolysis or acromial fractures in our study. In line with us, Baunach et al. revealed a low number of AC joint arthrosis, subacromial impingement, or rotator cuff tear with hook plate fixation [35]. Early removal of the plate following bone union may be crucial to preventing complications [35].

The only study that compared T-plate and hook plate directly by Tan et al. [11], showed that the T-plate group demonstrated greater improvements in activities of daily living, pain, and ROM, resulting in a higher rate of excellent and good results (UCLA score > 29) than the hook plate group (*P* = 0.001). When the hook plates were taken off, shoulder function improved greatly due to pain relief, and UCLA scores were equal to those of the T-plate group [11]. They did not nevertheless, their study had a retrospective design and had a low sample size which could affect the results. The material properties of locking plates also make them more expensive than non-locking ones, and developing countries have less access to them [36, 37]. However, we used a standard non-locking T-plate due to lower cost and accessibility in our developing country. Similar to our study, pain at the beginning limited ROM and function in the hook plate group, but at the last follow-up a similar score was reached as in the T-plate group. The pain can impair shoulder function, especially in the case of > 90° abduction.

The Tan et al. [11] study did not report any complications for the T-plate group. Additionally, we did not observe any complications in our study except for a fixation failure in the T-plate group, which was the same as in the hook plate group. In theory, T-plates are low-profile metals that do not rigidly fix fractures as hook plates do with their hooks. This could increase the risk of fixation failure and loosen in old-aged patients with compromised bone density. Future studies should test this hypothesis. In addition, T-plate fixation is impossible in patients with small distal fragments for insertion of 3 screws. Other complications seem to be similar to those observed in this study.

This study utilized a 3.5-mm standard non-locking T-plate that is typically used for distal radial fractures, but it provided fixation at this site by placing multiple screws in the small distal fragment. Using multiple screws and a higher angle of fixation improves fracture fixation, resulting in better grip, increased resistance to fracture extraction, and a more effective fixation [11, 38]. The T-plate does not enter the subacromial space and thus does not induce rotator cuff damage or impingement [17–19, 22, 39]. This technique induced lower cost than locking one [36, 37], and having no prior plan for plate removal surgery in the absence of symptoms made it more affordable than hook plate fixation [40]. It is crucial in developing countries such as ours that have limited access to expensive implants. According to a study by Fox et al. regarding the cost-effectiveness of several fixation methods for Neer 2 distal clavicle fractures, the double suture button technique is the most cost-effective method due to its low revision and complication rates [40]. A hook plate costs significantly more than suture buttons and locking plates from a healthcare perspective ($5,360 vs. $3,713 and $4,007 respectively) [40]. It was found that locking plates and suture buttons produced similar clinical results. This cost difference between hook plates and the two superior strategies is the result of the loss of productivity caused by hook plate removal [40].

This study faces serious limitations. First, the allocation to two groups was not random, making it susceptible to selection bias. Secondly, the follow-up period was limited to 6 months, and short follow-ups make it impossible to determine the long-term results of the two groups. However, the non-union in the clavicle is defined by 6 months of lack of healing [41], and all the participants showed complete union during the study period. Finally, the surgery was not preceded by MRI imaging, nor did we detect soft tissue and ligament damage during the procedure. Therefore, we cannot provide the type of fracture (IIA or IIB) in the participants. The strength of this study is the prospective follow-up without dropouts and the sufficient matched sample size.

## Conclusion

Based on the results of our study, both surgical approaches were associated with complete healing and good functional scores. However, in the hook plate group ROM and pain scores were lower at 1-month. The patients undergoing T-plate fixation did not need hardware removal within the first six postoperative months. Thus, it may have potential advantages to the hook plate that should be addressed in future investigations. Standard non-locking T-plates are a viable alternative to hook plates with low cost and promising outcomes for treating displaced distal clavicle fractures.

## Data Availability

Not applicable.
